# Don’t worry, be happy: Habitat selection of Blanding’s Turtles (*Emydoidea blandingii*) living in a reference condition in Georgian Bay

**DOI:** 10.1371/journal.pone.0295067

**Published:** 2023-12-13

**Authors:** Reta Lingrui Meng, Patricia Chow-Fraser

**Affiliations:** Department of Biology, McMaster University, Hamilton, Ontario, Canada; MARE – Marine and Environmental Sciences Centre, PORTUGAL

## Abstract

Few areas within the Great Lakes basin are currently free from impact of human activities, and it is important to study these reference conditions for comparison with degraded sites in those regions. Here, we use radio telemetry to investigate habitat use, movement, and habitat selection of a population of the endangered (Federally in Canada) Blanding’s turtle (*Emydoidea blandingii*, BLTU) inhabiting a mostly undisturbed archipelago located at the northern shore of Mnidoo gamii (Georgian Bay), Ontario over two active seasons (May to September 2021 and 2022). We found a mean home range of 16.21 ha for females (n = 7) and 15.10 ha for males (n = 7). Of the five habitat classes (Marsh, Open Water, Rock, Peatland, and Forest), females used all except Peatland during the nesting season, and both sexes used all five habitat classes throughout both active seasons in 2021 and 2022. Disproportionate habitat use was detected at the landscape scale but not at the home range scale which was consistent with the hypothesis that adult Blanding’s turtles residing in relatively undisturbed sites with abundant habitat types use all habitat types according to their availability. We also observed the use of open, deep water by Blanding’s Turtles as travel corridors for nesting and mating. Effective future conservation strategies should prioritize the protection and connectivity of relatively undisturbed wetlands, forests, and rock barrens in this region and use this study as a reference condition to compare BLTU habitat use and movement across disturbance gradients within Georgian Bay.

## Introduction

The Blanding’s turtle (*Emydoidea blandingii*, BLTU) is a North American semi-aquatic freshwater turtle species with a distribution centered around the Great Lakes provinces and states, extending north to the North Channel in Ontario, west to Cherry County Wetlands in Nebraska [1], south to the northern half of the state of Illinois [2], and east to the province of Quebec, with some smaller disjunct populations in New York, Massachusetts, Maine, and the most isolated population in Nova Scotia [3].The current conservation status of BLTU in jurisdictions throughout its geographic range vary from Immediate Concern (Pennsylvania), Special Concern (Wisconsin, Michigan, Ohio), Imperiled or Vulnerable (Nebraska Game and Parks), Threatened (Iowa, Minnesota, Ontario, Quebec, New York, Massachusetts) or Endangered (Indiana, Illinois, South Dakota, Missouri, Maine, New Hampshire, Nova Scotia). Like many other freshwater reptiles, the greatest threats to BLTU are habitat loss and road mortality [4], both of which are difficult to mitigate because of the turtle’s requirement for both terrestrial and aquatic habitats [5, 6] coupled with high site fidelity to nesting and brumation sites, even if the habitat has been modified or degraded [7, 8].

Many studies confirm the importance of a variety of aquatic and terrestrial habitats, particularly wetlands, as critical habitats for the semi-aquatic BLTU. Peatlands (fens and bogs) and marshes are important for mating, foraging, thermoregulation, and brumation; together with vernal pools in forests, these wetlands also provide refuge from predators while they move and travel during nesting (see Table 1). Yet, wetlands have been one of the most modified landscape features in North America following European colonization; there has been a net loss of >70% of wetlands in southern Ontario [9] and a net loss of 56% of wetlands across North America [10]. The Committee on the Status of Endangered Wildlife in Canada (COSEWIC) [3] estimated that over the past three generations, 60% of the Great Lakes/St. Lawrence BLTU population has been lost due to large-scale wetland conversion for agricultural and urban development, and a further decline of over 50% of the remaining populations is projected over the next three generations based on observed trends in road mortality. Two other important habitat types are for basking (rocks, fallen logs or floating vegetation mats) and nesting (sandy beaches, lichen/moss in rock crevices, or loose substrate by roadsides, gardens, and driveways) (Table 1).

**Table 1:** Definition of habitat classes used by the Blanding’s Turtle (*Emydoidea blandingii*) found within our northern Georgian Bay archipelago study site.

Habitat Class	Habitat Description	Life Cycle Activities	Relevant Literature
Peatland (Fen/Bog)	• Static or slow-flowing water, soft organic substrate, with emergent, floating, and/or submerged vegetation. • Abundance of basking sites, e.g., hummocks, shoreline, vegetation mats, emergent logs, and rocks	• Mating • Thermoregulation • Foraging • Aestivation/Resting • Travelling • Brumation	• COSEWIC, 2016 [3] • Markle and Chow-Fraser 2014 [13] • Joyal et al., 2001 [43] • Congdon et al. 2011 [51] • Edge et al. 2010 [6] • Seburn 2010 [46]
Marsh	• Unfrozen water, with soft organic substrate which is periodically or permanently flooded. • No trees with *Typha spp*. as dominant vegetation	• Brumation • Travelling • Summer inactivity • Thermoregulation • Mating • Foraging	• COSEWIC, 2016 [3] • Gillingwater and Brooks 2001 [47] • Congdon et al. 2011 [51] • Edge et al. 2010 [6] • Seburn 2010 [46]
Vernal Pools/Shallow water	• Small, shallow, ephemeral wetlands in forests • Transitional wetlands between larger bogs, swamps, etc.	• Travelling • Foraging • Temporary refugia	• Markle and Chow-Fraser 2014 [13] • Congdon and Keinath, 2006 [5] • Beaudry et al., 2009 [48]
Open Water	• Open water in the littoral zone	• Travelling • Brumation (rare)	• COSEWIC, 2016 [3] • OMNR, 2017 [52]
Rock Barren	• Exposed granitic rocks • Rock crevices • Little to no vegetation with abundant sunlight	• Nesting • Thermoregulation	• COSEWIC, 2016 [3] • Smolarz 2017 [49] • Markle and Chow-Fraser 2014 [13]
Forest	• Deciduous, mixed, and coniferous forests • Refuges including leaf litter and juniper plants. • Adjacent to wetlands	• Aestivation/Resting • Travelling • Foraging	• COSEWIC, 2016 [3] • Joyal et al. 2001 [43] • Congdon et al. 2011 [51] Refsnider and Linck, 2012 [44]

Habitat selection studies help determine patterns of resource and habitat use by animals and can help conservation efforts in determining where animals are more likely to occur [6]. This is particularly important for species at-risk that use a large variety of habitat classes such as the BLTU [6]. The extent to which BLTU populations use one habitat over another depends on several factors including availability of the habitat class on the landscape, accessibility of the habitat class to the turtle, and the importance of that habitat class to the turtle. Past studies have shown that BLTU do not generally use habitat classes at random but exhibit positive selection for wetlands and nesting habitats [11–13]. In more disturbed landscapes found throughout much of the BLTU’s species range and given the large variety of habitat classes that a BLTU will normally use during a year, there is a high probability that at least some required habitat classes in these areas would become limiting and, thus, lead to disproportionate use of habitat classes within the individual’s home range [6]. Human activities such as agricultural and urban development are also known to degrade wetlands [14, 15] and may lead to fragmentation of habitat patches of different quality. Therefore, in disturbed regions where a mosaic of habitat quality and types exist, BLTU may exhibit relatively high positive or negative selection for limited habitat types.

Another scenario is where BLTU populations exhibit neutral selection (neither positive nor negative) for available habitat classes because they live in primarily natural and undisturbed landscapes, or “reference conditions”, characterized as baseline habitats with abundant, high-quality resources with little to no threats to the target species so that it can survive and reproduce [16]. The concept of reference conditions stem from restoration ecology and is often used to help evaluate levels of degradation in other ecosystems, set restoration targets, and evaluate success of restoration [17]. Similarly, this concept can be applied to species at-risk conservation to help evaluate levels of threats in other populations, set conservation targets, and evaluate management success [16].

The objective of this study was to determine how BLTU residing in reference conditions in the coastal zone of Georgian Bay select habitat, particularly in a landscape with many islands, given that the world’s largest freshwater archipelago occurs in eastern Georgian Bay [18]. Our sub-population inhabits an archipelago at the northern tip of Georgian Bay, where both the level of threats and disturbances are low. Therefore, we hypothesize that BLTU in this archipelago will have abundant high-quality habitats to support their biological needs and would not exhibit positive or negative selection of any habitat classes. We compare the results in our study to ten other published studies on BLTU habitat use and selection in a gradient of disturbed to relatively pristine study areas. Our results will add to the limited knowledge on BLTU residing in undisturbed regions of Georgian Bay and provide a basis for understanding habitat selection for BLTUs living in reference conditions throughout its geographic range.

## Methods

### Study site

**Fig 1:**
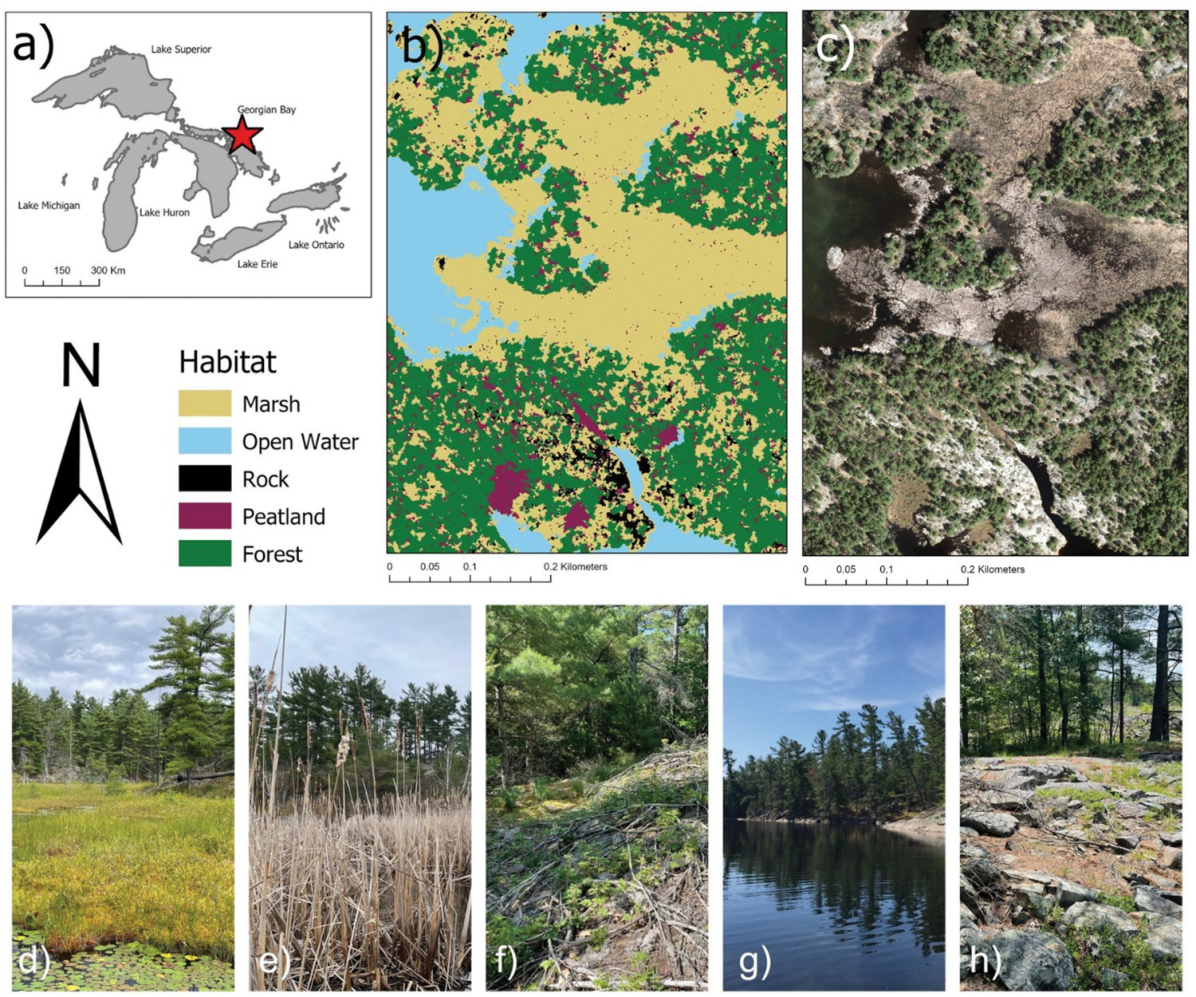
Location of population home range of Blanding’s Turtle in the northern Georgian Bay archipelago study site. A) The Great Lakes region, B) Closeup of the study site, which is one main island, and C) satellite imagery of the study site. Ground-level photos of habitat classes showing D) peatland, E) Marsh F) mixed deciduous-coniferous forest, G) open water, and H) rock barren.

We conducted our study in an isolated archipelago located along the northern shore of Mnidoo-gamii (Georgian Bay, The Great Lake of the Spirit, Fig 1). Georgian Bay is located at the northern range limit of the BLTU where there are still relatively abundant populations [19] and where coastal wetlands remain relatively undisturbed [20]. Georgian Bay is the eastern arm of Lake Huron with a landscape characterized by Canadian Shield bedrock, many small wetlands with shallow substrate, and thousands of islands with minimal human disturbance and low road density. Despite BLTU being consistently observed both historically and recently [19], there has been very little research on the ecology of Georgian Bay BLTUs. Management advice based on studies conducted in southern regions of BLTU range, where there are primarily fragmented wetlands, high road density, and high levels of human activities, are not appropriate for Georgian Bay. The archipelago study site is composed of approximately 500 islands ranging in size from large, inhabited islands (1125 ha) to small, rocky islands (0.006 ha). The region has minimal anthropogenic disturbances, with low human density and activity, largely limited to the summer months. Cottaging activities mainly consist of recreational boating and fishing. Indigenous peoples from the surrounding First Nations practice traditional harvesting and use the land year-round for hunting and snow machining. Our study area occurred on one main island with adjacent coastal cattail marshes connected to upland forest, rock barren, and open water (Fig 1). There are three main wetlands along the coast and upland of this island: A) a *Typha* spp. dominant coastal wetland, B) a coastal wetland with isolated *Typha* spp. strands accompanied by meadow sedge *Carex* spp. vegetation and *Gramineae* spp., C) a peatland wetland containing *Sphagnum* spp. and *Nepenthes* spp. plants situated along the edges of an inland lake (depth >2m). In this study, resident wetlands are functionally defined as the wetland where a turtle spends most of its time throughout its active season [13].

### Turtle tracking

**Fig 2:**
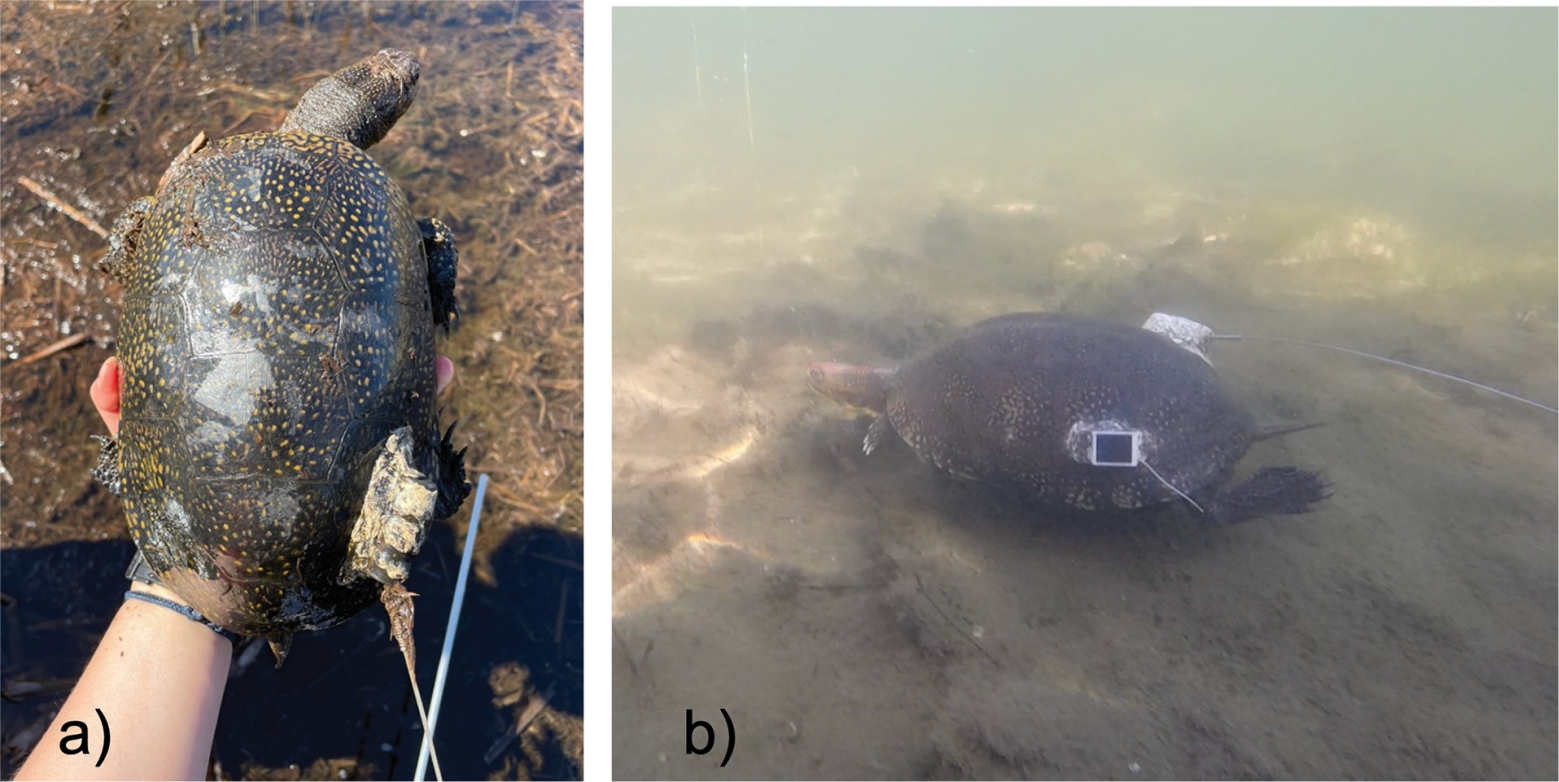
A Blanding’s Turtle. A) Shown with a VHF radio transmitter attached to its rear marginal scutes and another B) observed swimming in deeper, open water (~2m) within the archipelago. Also shown is a GPS logger attached on the left side of its rear marginal scutes.

We captured a total of 14 turtles during spring (May-June) of 2021 and 2022 (six and eight turtles, respectively) using baited hoop nets and hand capture. Upon capture, we determined sex using secondary morphological features (i.e., concavity of plastron, position of cloacal opening, and tail size). We weighed each individual to ensure the weight of our radio transmitters would be < 5% of their body mass. Once weight was confirmed, we attached AI-2F radio transmitters (Holohil Systems Ltd., Carp, ON, Canada, 19g; Fig 2A) to the rear marginal scutes with WaterWeld^TM^ epoxy (J-B Weld, Sulphur Springs, Texas, U.S.). We allowed the epoxy to set for 20 minutes before using a black marker to colour in the white appearance of the epoxy to lessen risks of predation. We notched the scutes of each turtle with a unique code for identification when recaptured [21]. We recorded any existing visible injuries and/or deformities and weighed each individual a second time to confirm that there were no large increases (>5% total body weight of individual) in total weight from transmitter attachment. We then released each turtle back to the location in which it was caught. We radio tracked six adult BLTUs (3 females, 3 males) between May 10^th^ and October 1^st^ in 2021 and 14 adult BLTUS (7 females, 7 males) between May 10^th^ and July 30^th^ in 2022 at a frequency of 2–3 times per week (±2). The individuals we captured in 2021 were tracked for two years. We also acquired additional data for females during nesting season in June of 2022 using GPS loggers (Fig 2B). The GPS loggers recorded location of turtles at four-hour intervals when they were above water, and the accuracy was ± three meters. GPS locations were excluded from data analysis for habitat selection and home range size and were used to determine nesting site locations as well as turtle behavior for a co-occurring project.

### Determining individual home range and movement

Using our relocation data, we calculated 100% Minimum Convex Polygons (MCP) [22] which represented home ranges of each BLTU in 2021, 2022 and mean home range across both years. The MCP method is widely used to estimate reptile home range [13, 23–25] and removes arbitrary choices in the selection of a smoothing factor [26]. We chose 100% MCPs to encompass all observed BLTU habitat use in this study site, and to allow comparisons with other studies. We conducted a Wilcoxon Matched-Pairs Signed Ranks Test (R version 4.1.2, R Core Team) to determine any significant differences between years for the home range size of both male and female BLTU (Turtle ID 1 to 6 that were tracked for both years). We also compiled both 2021 and 2022 home range values for each sex and conducted a Wilcoxon signed rank test to determine if there were significant differences between sexes across both years. We used non-parametric tests for statistical analysis due to the small sample size of 14 turtles in our study. We estimated Daily Distance Travelled (DDT) for BLTU in 2021, 2022, and mean DDT across both years from our radio telemetry data using the as.ltraj function on the adehabitatLT package in R (version 4.1.2, R Core Team). This function creates trajectories for individual turtles and creates an estimate for the mean movement per day in metres. We conducted a Matched-Pairs Signed Ranks nonparametric test given the small sample size to determine if there were significant differences for DDT values between sexes and between years. Results are considered significant if p-value <0.05.

### Habitat classification

We created a highly accurate habitat map to determine the proportion of habitat classes in our study site. We identified habitat classes using open-source orthophotos from the Central Ontario Orthophotography Project (COOP) captured between April 2^nd^—June 1^st^, 2021. COOP is a leaf-off, high-resolution imagery that is orthorectified. We used a mean-shift segmentation in ArcGIS Pro 2.0 (ESRI, Redlands, California) to segment our imagery, then used the ArcGIS Pro Classification Wizard to conduct supervised, object-based image analysis with a Support Vector Machine (SVM) algorithm. We mapped five main habitat classes including rock barren formed from Canadian Shield granitic bedrock, deciduous-coniferous mixed forests, cattail-dominated coastal marshes, peatland wetlands, and open water. We used the Canadian Wetland Classification System to identify wetlands as marsh and peatland [27]. We used both ground-truth points collected during our 2021 and 2022 field seasons and from visual interpretations of our high-resolution COOP imagery. We used a minimum of 30 training samples per class to train our SVM model (90 total ground truth points, 60 total visual reference points). We then assessed the accuracy of our classification model by generating random points using an equalized stratified random sampling strategy. Each random point was further visually interpreted in COOP high-resolution imagery to identify habitat type. In total, we used 30 ground truth points and 370 randomly generated points to assess the accuracy of our classification. Our habitat classification produced a high accuracy of 92.7% with a kappa coefficient of 0.901, allowing us to move forward with analysis using the produced habitat map.

### Macrohabitat selection

We investigated macrohabitat selection at two biologically relevant scales for the entire population and between sexes at the second order and third order scale. Second order is defined as selection of an individual’s home range from the entire population home range [6, 28, 29]. We determined the population home range by creating a MCP around all turtle relocations; then created home range kernels for each individual using a smoothing function [26]. We then followed methods outlined in Angoh et al. [29] to simulate available home ranges by creating 20 “available habitat kernels” for each turtle (n = 14, total of 280 simulated kernels) which allowed us to calculate and compare proportion of different habitats within each turtle’s kernel based on relocation data and the proportion of different habitats within simulated, randomly generated kernels. Third order habitat selection is defined as selection of an individual’s location from the individual’s home range [6, 28, 29]. We examined proportional use of habitat at this scale by following a similar method to Angoh et al.’s study [29] to compare the proportion of habitat types within each turtle’s habitat kernel and the proportion of habitat types at each relocation point. To determine whether there was disproportionate use of habitat by BLTUs within our site, we calculated Manly’s selection ratios [29–32]. Habitat selection is considered significantly positive or negative if selection ratios for habitat classes (± 95% confidence intervals; CI) do not overlap with 1. Positive selection (selection ratio > 1, ± CI) indicates that the habitat class is used more than its proportional availability, while negative selection (selection ratio < 1, ± CI) indicates that the habitat class is used less than its proportional availability. Habitat classes with selection ratios (± CI) that overlap with 1 are considered to have neutral selection, indicating that the animal is using the habitat proportional to its availability in the environment [29]. We validated our findings by conducting both compositional analysis and eigen analyses of selection ratios [29, 32, 33].

### Assembling metadata

We compiled 11 studies on adult BLTU habitat selection across the species’ range in both Canada and the U.S. We visually assessed the types of human activities present within each study site at the time of the study using Google Earth time-series imagery (studies range between 1990 to 2023). We used a list of 50 human disturbance classes identified to affect wetland ecological function in the literature [34, 35] and assessed for the presence of each activity within each study site (Table 2). We then classified disturbance types into four disturbance classes referencing Lomnicky et al. [34] and Herlihy et al. [35] (Table 3). We associated these disturbance types with each study site and compared habitat selection across a disturbance gradient based on the number of disturbance classes present at each site. We assume that more disturbance types also result in higher number of threats to BLTU within the site, as main threats affecting BLTU originate from anthropogenic activities [4].

**Table 2:** Type of disturbances found in ten published Blanding’s Turtle (*Emydoidea blandingii)* habitat use and selection studies conducted across the species’ geographic range in comparison to the current study site.

Study	Location	Disturbance types
Current Study	Northern Georgian Bay, Ontario	No visible disturbances
Edge et al. (2010)	Algonquin Provincial Park, Ontario, Canada	No visible disturbances
Bury and Germano (2003)	Nebraska Sandhills, U.S.	No visible disturbances
Markle and Chow-Fraser (2014)	Eastern shore of Georgian Bay in a national park, Canada	Docks
Hawkins MSc Thesis (2016)	Chalk River Laboratories, Canada	Industrial/Urban Buildings, Roads
Ross and Anderson (1990)	Petenwell Wildlife Area, Wisconsin, U.S.	Roads, Ditches
Joyal, McCollough, & Hunter (2001)	York County, Maine, U.S.	Suburban/Residential, Roads
Refsnider and Linck (2012)	Murphy-Hanrehan Park Reserve, Minnesota, U.S.	Fields, Pasture/Hay, Suburban Residential, Golf Courses
Angoh et al. (2021)	Northern Shore of Lake Erie, Ontario, Canada	Fields, Pasture/Hay, Suburban Residential, Roads, *Phragmites australis*
Chybowski et al. (2008)	Suburban/Rural Genesee, Wisconsin, U.S.	Fields, Pasture/Hay, Lawns, Suburban Residential, Parking Lots, Roads
Hartwig and Kiviat (2005)	New York, U.S.	Fields, Suburban Residential, Golf Courses, Roads, Wetland reconstruction

Land-use information was extracted from Google Earth timeseries imagery and literature description of each study site.

**Table 3:** Anthropogenic disturbance types found across Blanding’s Turtle (*Emydoidea blandingii*) species range consolidated into four disturbance classes for our study.

Disturbance Type	Disturbance Class
Fields	Agriculture
Pasture/hay	Agriculture
Industrial/Urban Buildings	Residential and Urban
Lawns	Residential and Urban
Suburban Residential	Residential and Urban
Golf Courses	Residential and Urban
Parking lots	Residential and Urban
Docks	Residential and Urban
Roads	Residential and Urban
*Phragmites australis*	Invasive Species
Ditches/Wetland Reconstruction	Hydrologic Modifications

Disturbance classes as related to BLTU habitat use assigned according to Lomnicky et al. [34] and Herlihy et al. [35] using Google Earth timeseries imagery and literature description of each study site. Each disturbance type is associated with a disturbance class.

### Ethics statement

Data collection was authorized by a McMaster University Animal Use Protocol (22-07-27), an Ontario Ministry of Natural Resources and Forestry Wildlife Scientific Collector’s Authorization (#1097649), and approval from the Ontario Ministry of Natural Resources and Forestry/Ministry of Environment, Conservation, and Parks Wildlife Animal Care Committee. Permits were renewed and active throughout the span of this study.

## Results

**Fig 3:**
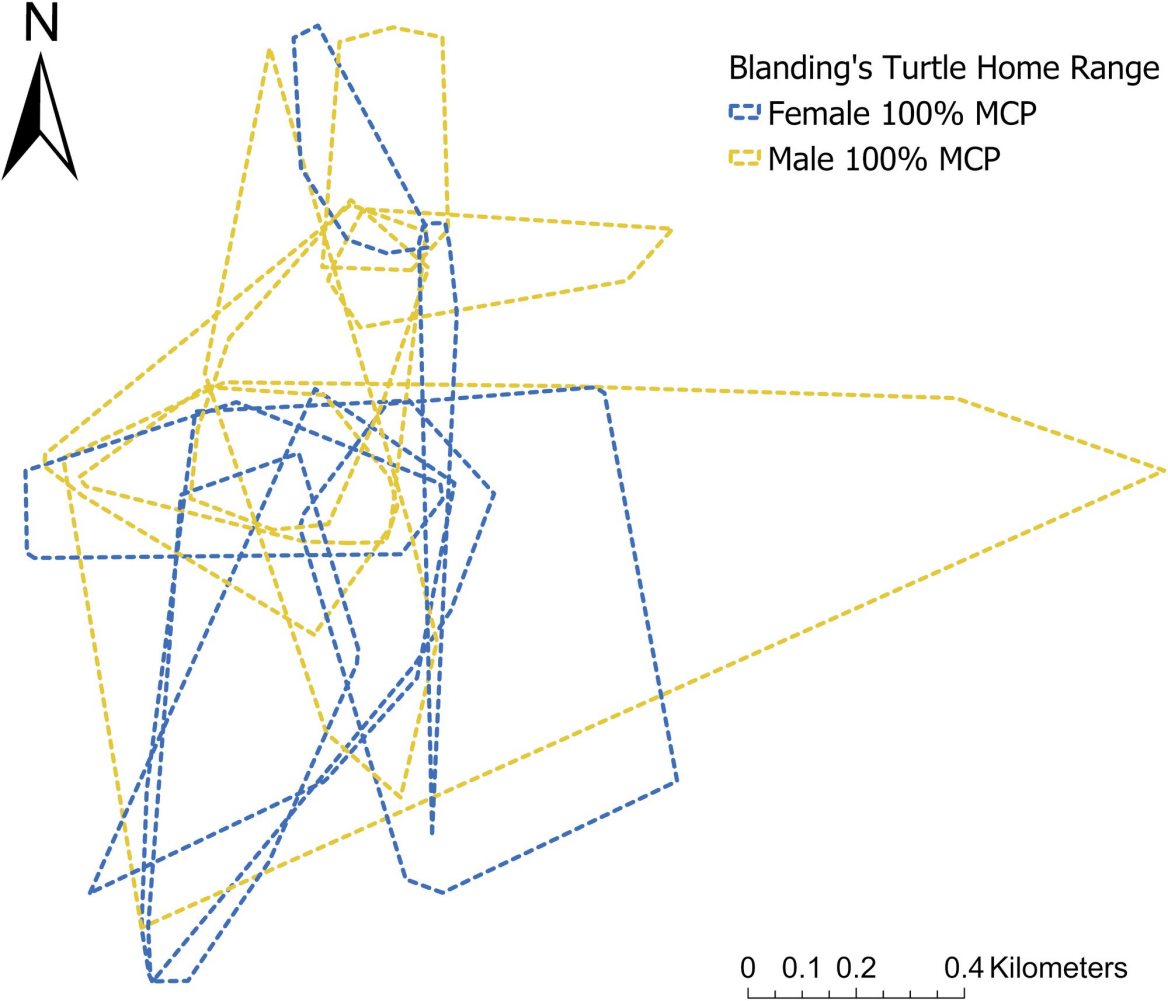
Map of 100% minimum convex polygon (MCP) of individual Blanding’s Turtle (*Emydoidea blandingii*) home ranges in the northern Georgian Bay archipelago study site. Each polygon represents the mean home range of an individual Blanding’s Turtle (n = 14) within the study area, created using 2021 and 2022 radio telemetry data.

Over the two active seasons, we relocated the 14 turtles 516 times (3X/wk) and determined the mean population home range for males to be 15.10 ha and for females to be 16.21 ha (Table 4; Fig 3). Given the large variation among individual home range for females (4.1 ha to 42.3 ha, SD = 12.8) and males (2.6 ha to 35.3 ha, SD = 10.4) we did not detect any significant differences between sexes (p-value > 0.05). Nor did we find any significant differences between years for each sex and for both years combined (Wilcoxon Matched-Pairs Signed Ranks Test; p-value > 0.05). Similarly, the mean DDT of 53.53 m/day for females and 55.04m/day for males were not significantly different between sex nor years (p-value > 0.05). This was true with or without including turtle 002, who migrated a long distance in July that increased his DDT values compared to other individuals (Table 5).

**Table 4:** Home range size of Blanding’s Turtles (*Emydoidea blandingii*) calculated using 100% minimum convex polygon method for 2021 and 2022 in our northern Georgian Bay archipelago study site.

**a) Female adult Blanding’s turtles**		
Turtle ID	Size (ha)	
	2021	2022
1	34.6	14.8
3	23.6	6.0
5	4.1	10.5
10		42.3
14		4.9
16		17.2
17		4.1
**b) Male adult Blanding’s turtles**		
Turtle ID	Size (ha)	
	2021	2022
2	12.9	35.3
4	2.6	10.5
6	9.6	26.1
11		27.5
12		14.6
13		2.9
15		9.0

Home range is estimated with 100% minimum convex polygons in 2021 and 2022 for female and male Blanding’s turtles living in the study site archipelago. Turtles were tracked between May 10^th^ and July 30^th^ in 2022 and between May 10^th^ and October 1^st^ in 2021.

**Table 5:** Daily Distance Travelled (DDT) calculated for Blanding’s Turtles (*Emydoidea blandingii*) in 2021 and 2022 in northern Georgian Bay archipelago study site.

**a) Female adult Blanding’s turtles**		
Turtle ID	Mean (±SE) DDT (m/day)	
	2021	2022
1	56.7 ± 14.3	52.9 ± 17.8
3	59.2 ± 23.3	48.4 ± 15.9
5	36.7 ± 12.4	45.3 ± 14.1
10		71.9 ± 27.0
14		38.3 ± 9.5
16		52.2 ± 8.5
17		73.7± 39.7
**b) Male adult Blanding’s turtles**		
Turtle ID	Mean (±SE) DDT (m/day)	
	2021	2022
2	55.8 ± 12.8	349.9 ± 254.0
4	32.3 ± 5.6	51.8 ± 12.8
6	60.3 ± 25.6	77.9 ± 20.0
11		91.7 ± 31.3
12		65.4 ± 19.5
13		25.4 ± 6.4
15		34.8 ± 7.3

DDT calculated for male and female Blanding’s turtles living in the study site archipelago during 2021 and 2022 using the adehabitatLT R package. Turtles were tracked between May 10^th^ and July 30^th^ in 2022 and between May 10^th^ and October 1^st^ in 2021.

We noted habitat use that included observations of BLTU regularly using deep, open water (> 2m depth, Fig 2B) to cross island boundaries and access resource patches during the active season. Deep water movement is especially apparent during the nesting season when three tagged female BLTUs accessed small islands adjacent to their resident wetland by crossing open water channels (200m ± 50m). The females made multiple nesting attempts and successfully nested on these adjacent small islands (with unconfirmed hatchling success). Two pairs of adult BLTU were also observed mating, and four adult BLTU were observed foraging in open water along island shorelines, which confirms that BLTU can use a variety of habitats including open water to fulfill life-cycle requirements at this study site.

BLTUs spent most of their time throughout their active season within three main resident wetlands, exiting only to complete nesting forays and forage for food (wetland A, B, and C). Nine individuals used wetland A as their resident wetlands, four individuals used wetland B as their resident wetland, and one individual used wetland C. Over the two years of study, BLTU displayed site fidelity to both their overwintering sites and resident wetlands. Five turtles used Wetland A for overwintering and one used wetland C for overwintering in both 2021 and 2022. No BLTU was tracked in Wetland B during 2021, and therefore, site fidelity was unconfirmed in this wetland. For overwintering in 2022, two individuals used a beaver pond (depth >2m, dominated by submerged and floating aquatic vegetation) located approximately 500m upland from Wetland A. Female BLTUs exhibited site fidelity to nesting locations across both years, and were observed to use rock barrens, forests, marsh, and open water during nesting season. Females did not appear to use peatlands during nesting season, but they may have returned to peatlands between nesting forays to regain moisture and were simply unobserved by research personnel due to the limitations in the frequency of observation.

**Fig 4:**
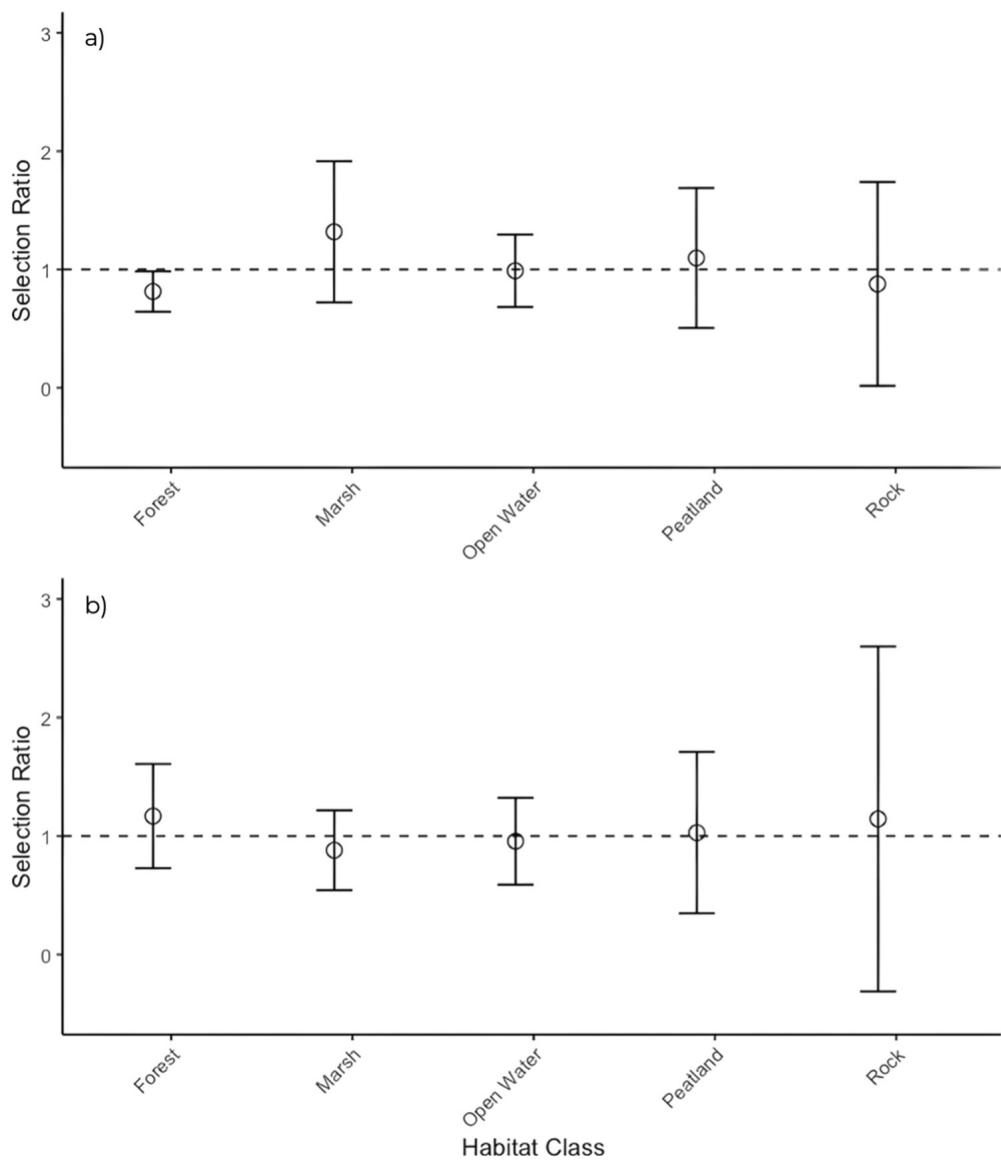
Manley’s selection ratio (± 95% CI) of habitat selection for 14 Blanding’s turtles. Habitat classes with both upper and lower confidence intervals above (positive selection) or below (negative selection) the selection ratio of 1 are considered to have significant selection. Habitat classes with selection ratios containing 1 are not showing positive nor negative selection. Habitat selection at the a) second-order, landscape scale and b) third-order, home-range scale.

**Fig 5:**
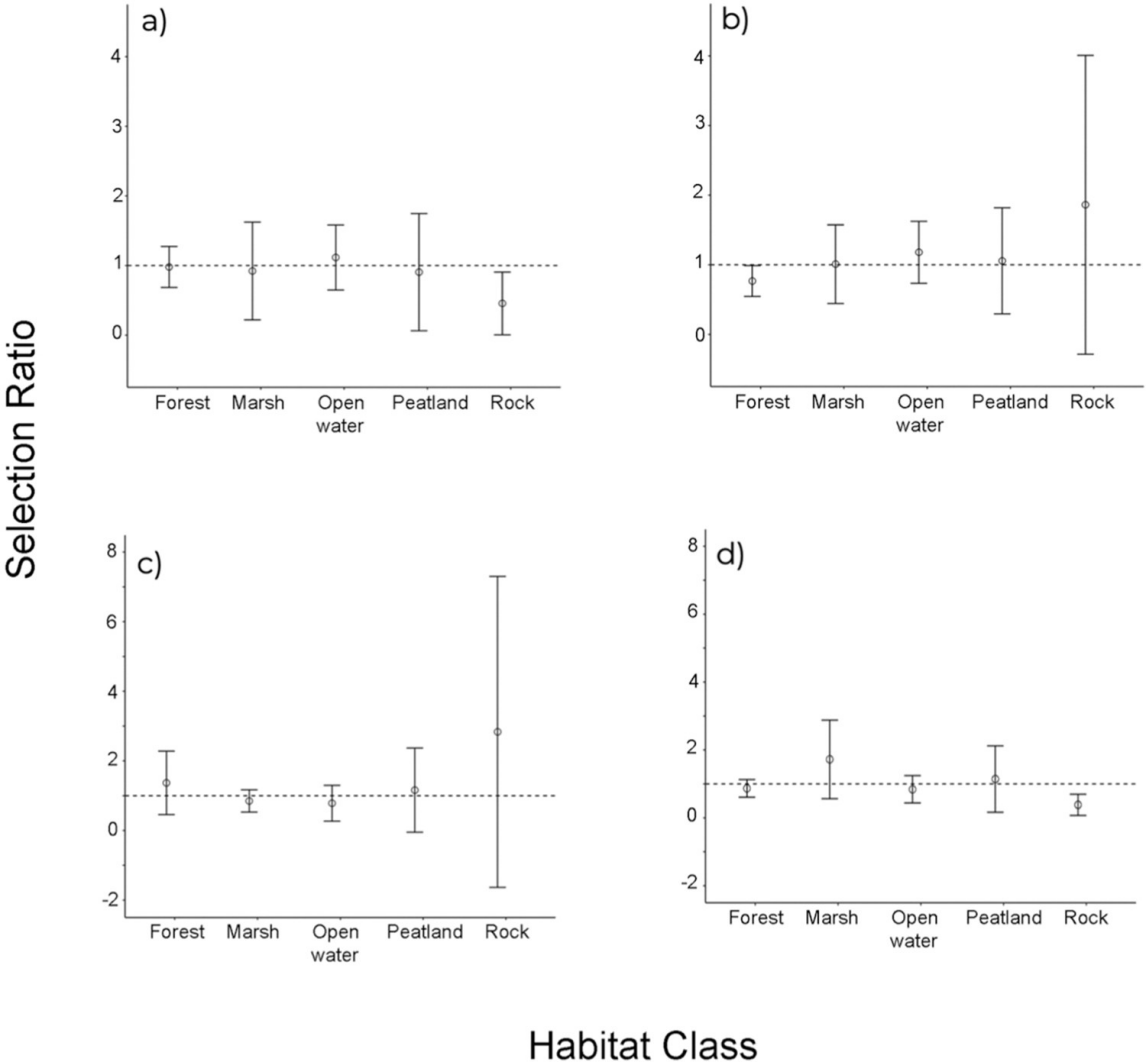
Manley’s selection ratio (± 95% CI) of habitat selection for male and female Blanding’s turtles. Habitat classes with both upper and lower confidence intervals above (positive selection) or below (negative selection) the selection ratio of 1 are considered to have significant selection. Habitat classes with selection ratios containing 1 are not showing positive nor negative selection. Habitat selection by a) male Blanding’s turtles at the third-order, home-rage scale (n = 7), b) male Blanding’s turtles at the second-order, landscape scale (n = 7), c) female Blanding’s turtles at the third-order, home-range scale (n = 7), and d) female Blanding’s turtles at the second-order, landscape scale (n = 7).

BLTU (n = 14) exhibited disproportionately lower use of upland forests at the second order, landscape level (CI for selection ratios <1.0; Fig 4A). At the third order, home range scale, BLTU did not exhibit disproportionate use of any of the five habitat classes (Fig 4B). We conducted analysis based on sex and found that males (n = 7) exhibited disproportionately lower use of forests at the third order scale (Fig 5A) and disproportionately lower use of rock barrens (usage < availability) at the second order scale (Fig 5B). Females (n = 7) exhibited disproportionately lower use of rock barrens (usage < availability) at the third order scale (Fig 5C) and did not demonstrate disproportionate selection at the second order scale (Fig 5D). Compositional analyses and eigen analyses of selection ratios produced similar results for habitat selection at both scales for the entire population and when separated by sex for males and females (Compositional analyses showed non-significant selection, whereas eigenvector analysis indicated no directionality of selection for habitat types by turtles).

Metadata compiled across 11 studies showed that when sites were undisturbed, BLTU did not exhibit disproportionate selection for most habitat types, as did the current study. As sites increased in level of disturbances (increased in number of disturbance classes), there was a shift towards disproportionate (negative or positive) habitat selection (Table 6). Not all highly disturbed study sites showed highly disproportionate selection across multiple habitat types, but these disturbed sites also contained very few natural habitat types and could not show positive or negative selection for habitat types that did not exist [36, 37].

**Table 6:** Comparison of habitat selection (negative (-), positive (+), neutral selection (0)) for habitat classes used by adult Blanding’s Turtles in ten published studies and the current study (Table 2).

Disturbance Class	Location	Order	F	R	M	B	T	S	P	O	A
None	Northern shore of Georgian Bay, Ontario, Canada (current study)	2^nd^ 3^rd^	**-** **0**	**0** **0**	**0** **0**	**0** **0**				**0** **0**	
None	Algonquin Provincial Park, Ontario, Canada [6]	2^nd^ 3^rd^	**-** **0**		**0** **0**	**0** **0**	**0** **0**	**0** **0**	**0** **0**	**0** **0**	
None	Nebraska Sandhills, U.S. [12]				**+**				**+**	**-**	
Residential/Urban	Eastern shore of Georgian Bay in a National Park, Canada [13]	2^nd^ 3^rd^	**+** **0**	**+** **0**	**0**	**+** **+**		**+** **0**		**0**	
Residential/Urban	Chalk River Laboratories Canada [11]	2^nd^ 3^rd^	**0** **-**		**+** **+**	**0** **0**	**0** **0**			**-** **0**	
Residential/Urban Hydrologic modification	Petenwell Wildlife Area, Wisconsin, U.S. [45]		**-**		**-**				**+**		
Residential/Urban	York County, Maine, U.S. [43]		**0**	**0**	**0**		**0**	**+**	**+**		
Agricultural Residential/Urban	Murphy-Hanrehan Park Reserve, Minnesota, U.S. [44]		**0**					**+**	**0**	**+**	**0**
Agricultural Residential/Urban Invasive Species	Northern Shore of Lake Erie, Ontario Canada [29]	2^nd^ 3^rd^	**0** **-**		**0** **0**					**0** **-**	**0** **-**
Agricultural Residential/Urban Invasive Species	Suburban/Rural Genesee, Wisconsin, U.S. [37]		**+**		**0**		**+**			**+**	
Agricultural Residential/Urban Hydrologic Modifications	New York, U.S. [36]				**+**			**+**			

Habitat selection is compared relative to number of disturbance classes (Table 3). F = Forest, R = Rock, M = Marsh, B = Bog, T = Thicket Swamp, S = Shallow water (vernal pool), P = Permanent pools/ponds, O = Open water/lakes and A = Agriculture/open land.

## Discussion

### Habitat selection

The identified archipelago BLTU population showed negative selection for forest habitats at the second-order scale and neutral selection for all habitat types at the third-order scale; however, negative selection at our study site is not likely a reflection of the poor quality of forest habitats but rather their abundance relative to other habitat types in the region. The proportional use of most habitat types at the second-order scale and all habitat types at the third-order scale aligns with our hypothesis that there are multiple suitable habitat types at our site and that BLTU are not strongly selecting for a particular habitat type when there is a range of suitable habitats available and when there are no barriers (roads or hydro corridors) to prevent access. This makes sense for BLTU as they have resource requirements that can be met by a wide range of habitat types throughout their active and non-active season. When compared to a similar, relatively undisturbed study site [6], BLTU in our study site exhibit comparable habitat selection.

### Establishing reference condition

We identified extensive and pristine habitat available to support all life stages and biological needs of BLTU within our study site. Currently, there are numerous basking locations in all three resident wetlands and abundant prey items that were visible during our surveys (presence of aquatic insects, Anurans, small fishes, etc.). The BLTU population appears to be healthy, evidenced by a diverse age distribution (based on radio tracked individuals and additional incidental sightings; n = 27) that includes juveniles (age 5–10), young adults (age 15–20), and mature adults (20+ years; age deduced from examination of growth rings on scutes). The presence of both adults and juveniles in a habitat suggest successful breeding and recruitment in BLTU populations [38]. All seven females within our study became gravid and nested successfully (based on approximate nesting locations from GPS loggers and assessment of gravid condition by palpation behind the hind legs). These are all positive indicators of a healthy population that suggest individuals are surviving to their physiological old age.

Additional factors supporting the presence of a healthy population is the high capture frequency since BLTU populations are often small and fragmented [3]. During incidental surveys between early May–mid-June in 2021 and 2022, new BLTU were regularly captured, with a notable capture event of three BLTUs together. There is no major threat to the turtles we captured because threats to other BLTU populations such as habitat loss, fragmentation, and road mortality [4] do not apply to this population. There are no roads in the archipelago, and cottage density is low, with a limited amount of developed land. We are also aware that BLTU have lived in this region for at least nine decades, as local cottagers showed us the shell of an adult BLTU that had been caught in the 1950s.

### Novel habitat use

Only a few studies have documented habitat use and selection of BLTU living in an archipelago [13, 39–41], and none have documented turtles travelling from island to island across a wide channel. BLTU are generally associated with use of shallow water (<2m) containing dense aquatic vegetation and organic substrate [3]. Previous studies also show that an increase in open water habitats decreased habitat suitability for BLTU but that open water could be used as travel corridors [42]. We observed one female crossing a 150-200m wide channel (>2m depth) to access nesting and mating resources. Nesting on isolated small islands surrounded by deeper water in this population may be a potential strategy to reduce predation risks, as mesopredators of BLTU nests (i.e., racoons, skunks, foxes) may not swim across deep water channels to access food sources, although nesting success remains unconfirmed.

### Metadata comparison

A survey of ten published studies on BLTU habitat selection and use showed that two studies were conducted in an undisturbed landscape (reference conditions), while the remainder were conducted in landscapes with a range of disturbance types (Table 2). Both reference sites, one in Nebraska [12] and the other in Algonquin Provincial Park [6], are located far from the coastal zone of Georgian Bay, and only the Algonquin Provincial Park study site contain comparable habitat types for BLTU to our study. The heterogeneous landscape from both the Algonquin Provincial Park study site [6] and this study include relatively natural and undisturbed habitat types, and the BLTU populations in both sites exhibited mostly neutral habitat selection. We also noticed a trend towards more positive or negative selection as sites increased in the type and number of disturbance classes, and this may be related to a general decline in the number of habitat classes available to turtles [36, 37, 43–45]. These observations support our hypothesis that adult Blanding’s turtles residing in relatively undisturbed sites with abundant habitat types use all habitat types according to their availability and will, therefore, show mostly neutral habitat selection. By comparison, turtles living in a disturbed landscape are more likely to exhibit positive or negative selection to the fewer habitat classes that are available to them.

Our population in northern Georgian Bay is unique in several ways. First, this population lives on islands in an archipelago. Secondly, the archipelago is located near the northern limit of its geographic range where both landscape and climate are different from those of populations residing inland or in southern Ontario and the midwestern U.S. Despite these differences, this population exhibited the same neutral selection for marshes, bogs and open water/lake as the population living in Algonquin Park more than 130 km from the coast. These similarities in habitat selection are likely because both study sites are in reference condition. We hypothesize that turtles will only show a positive selection for wetland habitats when some forms of human disturbances are present on the landscape (i.e., residential, urban, agricultural development). Our findings reinforce the importance of wetland for this species and emphasizes the need to include wetland restoration and protection, as well as ensuring connectivity among wetlands in all recovery plans. The BLTU is also considered an umbrella species for conservation, and by protecting this turtle species, we are also protecting many other co-occurring species-at-risk [50].
